# Spray-dried plasma attenuates inflammation and lethargic behaviors of pregnant mice caused by lipopolysaccharide

**DOI:** 10.1371/journal.pone.0203427

**Published:** 2018-09-12

**Authors:** Yanhong Liu, Jeehwan Choe, Jeong Jae Lee, Junsu Kim, Joy M. Campbell, Javier Polo, Joe D. Crenshaw, James E. Pettigrew, Minho Song

**Affiliations:** 1 Department of Animal Science, University of California, Davis, California, United States of America; 2 Department of Animal Science and Biotechnology, Chungnam National University, Daejeon, Republic of Korea; 3 APC Inc., Ankeny, Iowa, United States of America; 4 Department of Animal Sciences, University of Illinois, Urbana, Illinois, United States of America; CEFYBO (CONIET-UBA), ARGENTINA

## Abstract

This study evaluated whether dietary spray-dried plasma (SDP) can ameliorate inflammation, lethargic behaviors, and impairment of reproduction caused by lipopolysaccharide (LPS) challenge during late pregnancy. Two experiments were conducted with 125 mated female mice (C57BL/6 strain) in each experiment. All mice were shipped from a vendor on the gestation day (GD) 1 and arrived at the laboratory on GD 3. Mice were randomly assigned to dietary treatments with or without 8% SDP in the diet. On GD 17, mice determined pregnant by BW and abdomen shape were randomly assigned to intraperitoneal injections with or without 2 μg LPS. In experiment 1, 17 mice (26.7 ± 1.7 g BW) were identified pregnant and euthanized 6 h after the LPS challenge to measure inflammatory responses in uterus and placenta. In experiment 2, 44 mice (26.0 ± 1.6 g BW) were identified pregnant and euthanized 24 h after the LPS challenge to assess behavior and late-term pregnancy loss. Growth performance and reproductive responses, such as loss of pregnancy, percentage of fetal death, and etc., were measured in all pregnant mice. The LPS challenge increased (*P* < 0.05) uterine and placental tumor necrosis factor-α and interferon-γ, late-term pregnancy loss, and lethargy score, and decreased (*P* < 0.05) uterine transforming growth factor-β1, moving time and number of rearing, and growth and feed intake. The SDP decreased (*P* < 0.05) concentrations of both pro-inflammatory and anti-inflammatory cytokines in one or both tissues, and the lethargy score, and increased (*P* < 0.05) moving time and number of rearing, growth of pregnant mice, and fetal weight. However, the SDP did not affect late-term pregnancy loss caused by the LPS challenge. Consequently, dietary SDP attenuated acute inflammation and lethargic behaviors of pregnant mice caused by the LPS challenge, but did not affect late-term pregnancy loss after the acute inflammation.

## Introduction

Several studies have shown that acute inflammation during late pregnancy can cause fetal death, fetal growth retardation, and pregnancy loss [1, 2, 3]. Furthermore, suppression of pro-inflammatory cytokines by administration of an anti-inflammatory cytokine [2, 3], interleukin-10 (IL-10), or some specific components [4, 5, 6], attenuated those reproductive losses. Spray-dried plasma (SDP) has generally recognized anti-inflammatory effects. There is now accumulating evidence that SDP in diets of lactating sows improves their subsequent reproductive performance, including interval from weaning to estrus and farrowing rate, especially during summer months associated with heat stress [7, 8, 9]. Moreover, our research team found that feeding SDP to stressed mice shortly after mating increased pregnancy rate [10]. However, the connection between SDP and late pregnancy under acute inflammatory conditions has not been explored. So, we hypothesized that inclusion of SDP in the diet may attenuate fetal death, fetal growth retardation, and pregnancy loss.

Spray-dried plasma is often fed to newly weaned pigs because it increases growth rate and appears to protect the pigs from infectious disease [11, 12]. Although the mechanisms through which SDP provides physiological benefits are not fully understood [11], the proposed benefits may derive from immuno-modulatory effects [13, 14, 15], improvement of intestinal barrier function [12, 16, 17], or both.

Therefore, the objective of this study was to evaluate a potential benefit of SDP for improving reproductive performance by limiting inflammatory damage to reproductive performance during late gestation, in this case caused by lipopolysaccharide (LPS) challenge.

## Materials and methods

The protocol for this experiment was reviewed and approved by the Institutional Animal Care and Use Committee of the University of Illinois at Urbana-Champaign. The experiment was conducted in the mouse facility of the Institute for Genomic Biology building at the University of Illinois at Urbana-Champaign.

### Animals, housing, diets, and experimental design

Two experiments were conducted in order to measure inflammatory responses (Experiment 1) and pregnancy maintenance (Experiment 2). For each study, a total of 125 mated female mice (C57BL/6 strain; 16.0 ± 1.2 g BW) were shipped from a vendor (The Jackson Laboratory, Bar Harbor, ME, USA) to the university facility (Urbana, IL, USA) on the day when the vaginal plug was found (gestation day [GD] 1), arriving at the university facility on GD 3 after 2 d transport by air and ground. When the mice arrived at the facility, they were weighed and housed in individual cages with controlled temperature (23°C), humidity (40%), and a 12 h light and dark cycle. They were immediately randomly assigned to dietary treatments with or without 8% SDP in the diet (SDP or CON; 98 and 27 mated mice for CON and SDP, respectively, in the experiment 1; 62 and 63 mated mice for CON and SDP, respectively, in the experiment 2) and allowed free access to feed and water for 15 d. The level of SDP (8%) in the diet was determined based on the previous studies using rat or mouse [18, 19, 20, 21]. The diets were formulated to meet or exceed estimates of nutrient requirements of mice [22] and to have similar metabolizable energy, crude protein, and amino acids levels, and no antibiotics (Table 1). The diets were pelleted without heating (cold-pelleted) using a pellet press. The SDP was produced from bovine blood (APC, Inc., Ankeny, IA, USA).

**Table 1:** Ingredient composition of experimental diets.

	Dietary treatments^1^	
Item	CON	SDP
Ingredients, %		
Dried skim milk	53.10	33.68
Corn starch	19.90	31.25
Sucrose	10.00	10.00
Spray-dried plasma^2^	-	8.00
Soybean oil	7.00	7.00
Cellulose	5.00	5.00
AIN-93 MX^3^	3.50	3.50
AIN-93 VX^4^	1.00	1.00
DL-methionine	0.25	0.32
Choline bitartrate	0.25	0.25
Calculated energy and nutrient levels		
ME, kcal/kg	3483	3558
Crude protein, %	18.28	18.00
Ash, %	4.44	3.57
Ca, %	1.18	0.94
P, %	0.70	0.64

^1^CON = control diet; SDP = 8% spray-dried plasma diet.

^2^The SDP was produced from bovine blood (AP 920; APC, Inc., Ankeny, IA).

^3^Dyets, Inc., Bethlehem, PA. Provided as milligrams per kilogram of diet: calcium, 5,000; phosphorus, 1,561; potassium, 3,600; sodium, 1,019; chloride, 1,571; sulfur, 300; magnesium, 507; iron, 35; copper, 6; manganese, 10; zinc, 30; chromium, 1; iodine, 0.2; selenium, 0.15; fluorine, 1; cobalt, 0.5; molybdenum, 0.15; silicon, 5; nickel, 0.5; lithium, 0.1; vanadium, 0.1.

^4^Dyets, Inc., Bethlehem, PA. Provided per kilogram of diet: thiamin HCl, 6 mg; riboflavin, 6 mg;pyridoxine HCl, 7 mg; niacin, 30 mg; calcium pantothenate, 16 mg; folic acid, 2 mg; biotin, 0.2 mg; cyanocobalamin (vitamin B_12_), 25 μg; vitamin A palmitate, 4,000 IU; vitamin E acetate, 75 IU; vitamin D_3_, 1,000 IU; vitamin K_1_, 0.75 mg.

On GD 17, the mice BW were weighed and pregnancy of each mouse was determined on the basis of BW and shape of abdomen (normal abdomen of non-pregnant mice vs. full, bulgy, rough, or bumpy abdomen of pregnant mice). A total of 17 mice (26.7 ± 1.7 g BW; Experiment 1) for the measurement of inflammatory responses and 44 mice (26.0 ± 1.6 g BW; Experiment 2) for the measurement of maintenance of pregnancy were determined as pregnant. The experimental model used in this research created a very low pregnancy rate on CON (17 pregnant mice out of 160 mated mice), but a substantially higher pregnancy rate on SDP (44 pregnant mice out of 90 mated mice), as described in a companion paper [10]. The result was that more pregnant mice were available on SDP than on CON, in spite of originally assigning more mice to CON.

The pregnant mice were randomly assigned within study and dietary treatment to intraperitoneal injections with 200 μL PBS or with 2 μg LPS from *Salmonella enterica* (serotype Typhimurium; Sigma-Aldrich, St. Louis, MO, USA) in 200 μL PBS. The dose of LPS was determined by preliminary experiments (data are not presented) guided by the report of Robertson et al. [2]. The mice were euthanized by cervical dislocation under CO_2_ anesthesia 6 h after the LPS injection for measurement of inflammatory responses (Experiment 1) and 24 h after the LPS injection for maintenance of pregnancy (Experiment 2), respectively. Fetal and placental weights, growth performance, and organ weights were measured in both experiments 1 and 2.

### Measurements and sample collection

Measurements were growth performance (BW gain, feed intake, and calculated ratio between BW gain and feed intake [G:F ratio]), late-term pregnancy loss, number of live and dead fetuses, average live fetal and placental weight, and organ weight (liver, spleen, and lung). The total numbers of live and dead fetuses were determined by checking movement of each fetus immediately after opening the abdominal cavity and then live and dead fetuses, placentae, and organs were collected and weighed. The average fetal to placental weight ratio, and organ weight as a percentage of BW were calculated. In addition, gestational tissues (uterus and placenta) were collected from the pregnant mice euthanized at 6 h after the injection challenge, snap-frozen in liquid nitrogen, and stored at -80°C until cytokine and total protein measurements. These gestational tissues were chosen for measurements based on a previous study [2].

### Cytokine and protein analyses

The processing of gestational tissue samples was based on the report of Robertson et al. [2]. The frozen uterus and placental samples were weighed, chopped by scissors, placed in conical tubes, and then cold protease inhibitor (5 mL/g sample; Roche Diagnostics, Indianapolis, IN, USA) dissolved in PBS (1 tablet [3.7 mg protease inhibitor]/7 mL PBS) was added. The samples were homogenized for 45 or 30 s for uterus or placental samples on ice, respectively, using a high-speed homogenizer (Fisher Scientific, Pittsburgh, PA, USA). Tissue lysates were centrifuged at 10,000 × g for 20 min at 4°C and supernatants were collected and stored at -80°C for cytokine measurements. Each cytokine was measured using mouse ELISA kits following the instruction of manufacturer (tumor necrosis factor-α [TNF-α; Invitrogen Corporation, Grand Island, NY, USA]; interferon-γ [IFN-γ; R&D systems, Minneapolis, MN, USA]; Interleukin-10 [IL-10; Invitrogen Corporation, Grand Island, NY, USA]; transforming growth factor-β1 [TGF-β1, Invitrogen Corporation, Grand Island, NY, USA]). Total protein (TP) concentration in the tissue lysates and bovine serum albumin as a standard were measured by the Bradford protein assay (Bio-Rad Laboratories, Hercules, CA, USA), and the data were used to normalize the cytokine concentrations. All the cytokine measurements of gestational tissue samples were based on the report of Robertson et al. [2]. The intra-assay coefficients of variation for TNF-α, IFN- γ, IL-10, and TGF-β1 were 5.9, 4.9, 6.5, and 5.7%, respectively. The inter-assay coefficients of variation for TNF-α, IFN- γ, IL-10, and TGF-β1 were 8.7, 8.3, 9.4, and 6.6%, respectively. All data for cytokine measurements were expressed as pg or ng cytokine/mg TP.

### Behavior observation

In experiment 2, all pregnant mice were monitored at 24 h after the LPS injection by 2 independent evaluators with the methods of Tuli et al. [23], van Ruivan et al. [24], and Kiank et al. [25] with slight modification. The mice were observed during 5 min after a major stimulus, consisting of moving the individual cage from one place to another in a rack of cages. Behavioral measurements included locomotor activity, including time of moving and number of rearing from four paws to two paws, and score for activity and exploration from 0 to 6 (lethargy score; 0 = normal activity, 1 = slightly reduced exploration, 2 = reduced exploration with short intervals without activity, 3 = longer intervals without activity, 4 = strongly reduced activity and huddling, 5 = huddling and lethargy but reaction to environmental stimulus, and 6 = no reaction to environmental stimulus). The behavior observations were measured before (0 h) and at 3, 6, 12, and 18 h after the injection challenge.

### Statistical analyses

Data were analyzed as a completely randomized design with a 2 × 2 factorial arrangement of treatments using the PROC GLM of SAS (SAS Inst. Inc., Carry, NC, USA). The experimental unit was pregnant mouse and litter. For growth performance, number of total, live, and dead fetuses, fetal death, average fetal to placental weight ratio, organ weight, and all cytokines, the statistical model included the effects of diet, injection challenge, and their interaction. The statistical model for behavior observations included the effects of diet, injection challenge, time, and their interaction. In addition, pair-wise comparisons were performed between dietary treatments when the main effect of diet was found. Late-term pregnancy loss after the injection challenge was analyzed by the χ2 test. Statistical significance and tendency were considered at *P* < 0.05 and 0.05 ≤ *P* < 0.10, respectively.

## Results

### Uterine and placental cytokines

**Fig 1:**
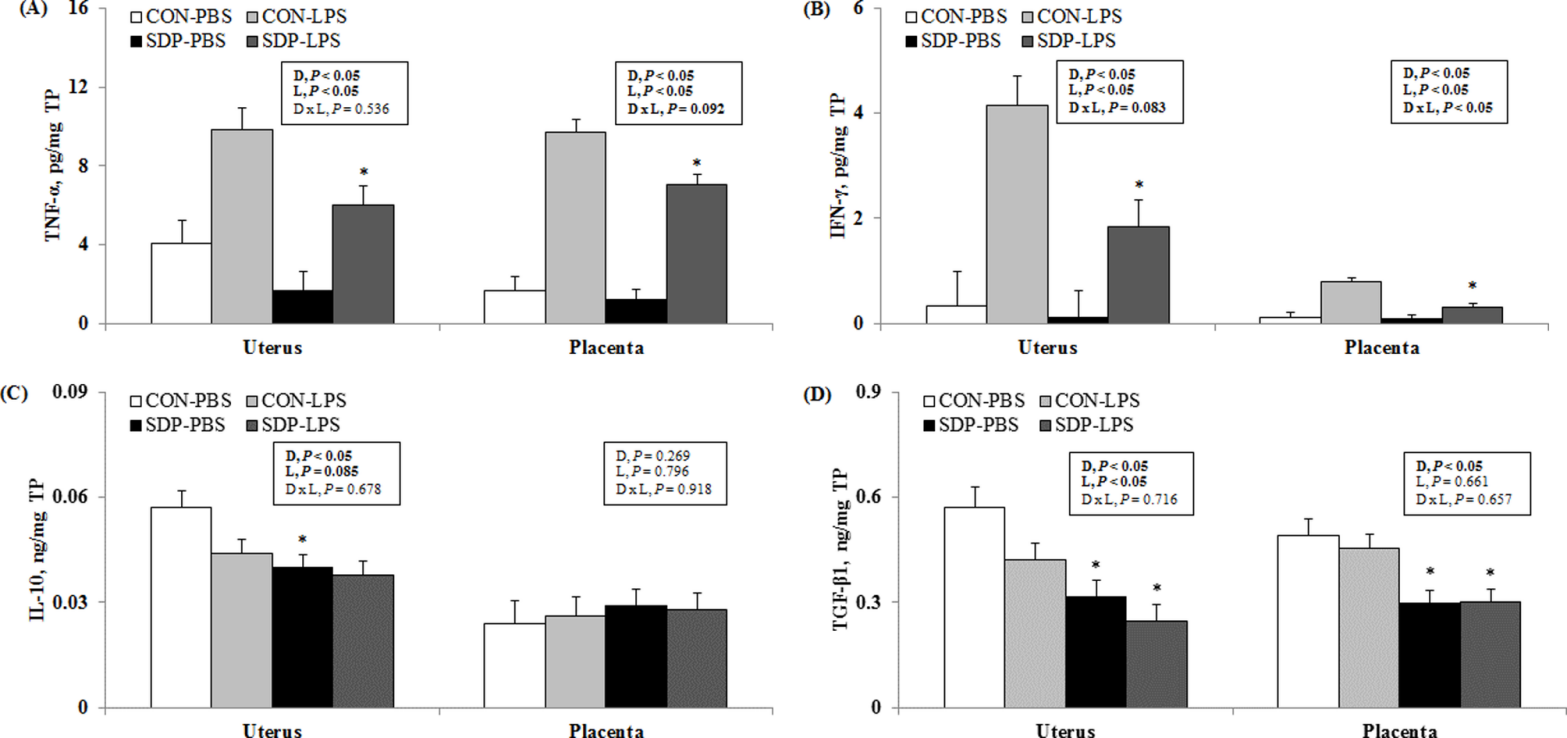
**Pro-inflammatory (A and B) and anti-inflammatory (C and D) cytokines in gestational tissues of pregnant mice as affected by dietary SDP and LPS challenge.** Values are means ± SE, *n* = 3, 4, 5, and 5 for CON-PBS (control diet + intraperitoneal injection of phosphate-buffered saline on GD 17), CON-LPS (control diet + intraperitoneal injection of lipopolysaccharides on GD 17), SDP-PBS (8% spray-dried plasma diet + intraperitoneal injection of phosphate-buffered saline on GD 17), and SDP-LPS (8% spray-dried plasma diet + intraperitoneal injection of lipopolysaccharide on GD 17), respectively. D, diet; L, LPS; D × L, interaction between diet and LPS. *Different from CON within a challenge treatment, *P* < 0.05.

The LPS challenge increased (*P* < 0.05) the concentrations of pro-inflammatory cytokines (TNF-α and IFN-γ; Fig 1[A] and 1[B], respectively) in both uterus and placenta and reduced the concentrations of anti-inflammatory cytokines (IL-10, *P* = 0.085; TGF-β1, *P* < 0.05; Fig 1[C] and 1[D], respectively) in the uterus only compared with the PBS challenge. The SDP reduced (*P* < 0.05) the pro-inflammatory effect of LPS in the concentrations of pro-inflammatory cytokines (TNF-α and IFN-γ) in both uterus and placenta compared with the CON. The effect of SDP on IFN-γ in placenta was greater in the presence of LPS (interaction *P* < 0.05). The SDP also reduced (*P* < 0.05) the concentrations of anti-inflammatory cytokines (IL-10 and TGF-β1) in uterus and of TGF-β1 in placenta compared with the CON. There were no treatment effects on the concentrations of placental IL-10 cytokine.

### Behavior

**Fig 2:**
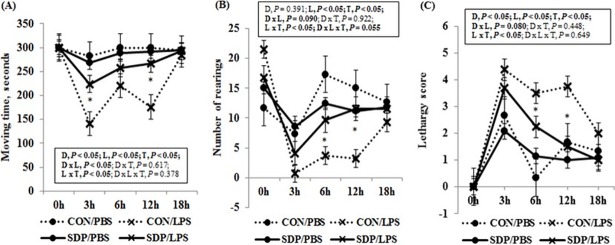
Behaviors of pregnant mice under acute inflammation by LPS challenge during 5 min. Values are means ± SE, *n* = 3, 4, 13, and 10 for CON-PBS (control diet + intraperitoneal injection of phosphate-buffered saline on GD 17), CON-LPS (control diet + intraperitoneal injection of lipopolysaccharides on GD 17), SDP-PBS (8% spray-dried plasma diet + intraperitoneal injection of phosphate-buffered saline on GD 17), and SDP-LPS (8% spray-dried plasma diet + intraperitoneal injection of lipopolysaccharide on GD 17), respectively. (A) Moving time, (B) Number of rearing, (C) Lethargy score (0 = normal activity, 1 = slightly reduced exploration, 2 = reduced exploration with short intervals without activity, 3 = longer intervals without activity, 4 = strongly reduced activity and huddling, 5 = huddling and lethargy but reaction to environmental stimulus, and 6 = no reaction to environmental stimulus). D, diet; L, LPS; T, time; D × L, interaction between diet and LPS; D × T, interaction between diet and time; L × T, interaction between LPS and time; D × L × T, interaction among diet, LPS, and time. *Different from CON within a challenge treatment at each time point, *P* < 0.05.

The LPS challenge depressed activity as it decreased (*P* < 0.05) the moving time (Fig 2[A]) and number of rearing (Fig 2[B]), and increased (*P* < 0.05) the lethargy score (Fig 2[C]) compared with the PBS challenge. The SDP attenuated those effects of LPS as it increased (*P* < 0.05) the overall moving time (Fig 2[A]) and number of rearing at some measurement times (Fig 2[B]), and decreased (*P* < 0.05) the overall lethargy score (Fig 2[C]) compared with the CON. The clearest effects of SDP on behavior were found between 3 and 12 hours after the LPS challenge. The interaction between diet and LPS reached significance (*P* < 0.05) in moving time.

### Reproductive performance

Reproductive performance was measured in both experiments. In experiment 1, either LPS challenge or dietary SDP did not affect the loss of pregnancy or fetuses in the pregnant mice (Table 2). In experiment 2, the LPS challenge increased (*P* < 0.05) the pregnancy loss and tended (*P* = 0.082) to increase the fetal death at 24 h after the LPS challenge (Table 2) compared with the PBS challenge. However, the SDP did not affect the late-term pregnancy loss and fetal death at 24 h after the LPS challenge compared with the CON.

**Table 2:** Maintenance of pregnancy and fetal death during 24 h after the LPS challenge^1^,^2^.

Item	Treatment^3^				*P*-value^4^		
	CON		SDP				
	PBS	LPS	PBS	LPS	D	L	D × L
During 6 h after LPS challenge							
All pregnant mice, n	3	4	5	5			
Pregnancy loss, n	0	0	0	0			
Pregnancy loss, %	0	0	0	0			
Mice that maintained pregnancy, n	3	4	5	5			
Number of fetuses/ litter	8.0 ± 0.8	7.3 ± 0.7	6.5 ± 0.7	7.8 ± 0.6	0.472	0.728	0.175
Number of live fetuses/ litter	8.0 ± 0.8	7.3 ± 0.7	6.5 ± 0.7	7.8 ± 0.6	0.472	0.728	0.175
Number of dead fetuses/ litter	0	0	0	0			
Fetal death, %	0	0	0	0			
During 24 h after LPS challenge							
All pregnant mice, n	3	7	13	21			
Pregnancy loss, n	0	3	0	11			
Pregnancy loss^5^, %	0	42.9	0	52.4	0.918	< 0.05	0.113
Mice that maintained pregnancy, n	3	4	13	10			
Number of fetuses/ litter	5.7 ± 0.9	7.0 ± 0.8	6.5 ± 0.4	6.8 ± 0.6	0.659	0.279	0.429
Number of live fetuses/ litter	5.7 ± 0.9	6.5 ± 0.8	6.5 ± 0.4	6.5 ± 0.6	0.585	0.549	0.585
Number of dead fetuses/ litter	0.0 ± 0.0	0.5 ± 0.2	0.1 ± 0.1	0.3 ± 0.1	0.609	0.056	0.338
Fetal death, %	0.0 ± 0.0	6.7 ± 2.9	1.3 ± 1.6	3.9 ± 2.0	0.765	0.082	0.429

^1^Values are means ± SE.

^2^There was no pregnancy loss among the pregnant mice euthanized during 6 h after the LPS challenge.

^3^CON = control diet; SDP = 8% spray-dried plasma diet; PBS = intraperitoneal injection of phosphate-buffered saline on GD 17; LPS = intraperitoneal injection of lipopolysaccharide on GD 17.

^4^D = diet effect; L = LPS challenge effect; D × L = interaction between diet and LPS challenge.

^5^Pregnancy loss during 24 h after injection was analyzed by the χ2 test.

The LPS challenge did not affect the average fetal and placental weights at 6 h after the LPS challenge, but tended (*P* = 0.079) to decrease the placental weight at 24 h after the LPS challenge compared with the PBS challenge (Table 3). The SDP increased (*P* < 0.05) the average fetal weight at 6 h after the LPS challenge and tended (*P* = 0.082) to increase it at 24 h after the LPS challenge compared with the CON. In addition, the SDP tended (*P* = 0.072) to increase the average fetal to placental weight ratio at 6 h after the LPS challenge compared with the CON.

**Table 3:** Fetal and placental weights^1^.

Item	Treatment^2^				*P*-value^3^		
	CON		SDP				
	PBS	LPS	PBS	LPS	D	L	D × L
At 6 h after LPS challenge, n	3	4	5	5			
Average fetal wt, g	0.577 ± 0.039	0.537 ± 0.030	0.660 ± 0.034	0.634 ± 0.034*	< 0.05	0.381	0.845
Average placental wt, g	0.113 ± 0.009	0.119 ± 0.007	0.112 ± 0.008	0.104 ± 0.008	0.354	0.921	0.435
Ratio^4^, g/g	5.13 ± 0.70	4.60 ± 0.55	6.06 ± 0.61	6.20 ± 0.61*	0.072	0.768	0.609
At 24 h after LPS challenge, n	3	4	13	10			
Average fetal wt, g	0.719 ± 0.024	0.697 ± 0.019	0.767 ± 0.018	0.752 ± 0.019*	0.082	0.544	0.911
Average placental wt, g	0.120 ± 0.009	0.103 ± 0.007	0.113 ± 0.004	0.103 ± 0.006	0.603	0.079	0.630
Ratio^4^, g/g	6.01 ± 0.76	6.76 ± 0.66	6.99 ± 0.37	7.46 ± 0.47	0.166	0.308	0.813

*Different from CON within a challenge treatment, *P* < 0.05.

^1^Values are means ± SE.

^2^CON = control diet; SDP = 8% spray-dried plasma diet; PBS = intraperitoneal injection of phosphate-buffered saline on GD 17; LPS = intraperitoneal injection of lipopolysaccharide on GD 17.

^3^D = diet effect; L = LPS challenge effect; D × L = interaction between diet and LPS challenge.

^4^Ratio = fetal weight divided by placental weight.

### Growth performance

The LPS challenge tended (*P* = 0.062) to decrease the BW gain and decreased (*P* < 0.05) the feed intake during 6 h after the LPS challenge, and decreased (*P* < 0.05) the BW gain, feed intake, and G:F ratio during 24 h after the LPS challenge compared with the PBS challenge (Table 4). The SDP tended (*P* = 0.057) to increase the BW gain during 6 h after the LPS challenge and increased (*P* < 0.05) the BW gain and G:F ratio during 24 h after the LPS challenge compared with the CON.

**Table 4:** Growth performance of pregnant mice^1^.

Item	Treatment^2^				*P*-value^3^		
	CON		SDP				
	PBS	LPS	PBS	LPS	D	L	D × L
During 6 h after LPS challenge, n	3	4	5	5			
BW, g	26.70 ± 0.80	25.20 ± 0.78	27.28 ± 0.76	27.28 ± 0.76	0.12	0.36	0.36
BW gain, g	0.00 ± 0.12	-0.28 ± 0.10	0.25 ± 0.09	0.00 ± 0.09*	0.057	0.062	0.924
Feed intake, g	0.10 ± 0.06	0.03 ± 0.05	0.30 ± 0.05*	0.10 ± 0.05	< 0.05	< 0.05	0.249
G:F ratio^4^,^5^, g/g	-	-	-	-	-	-	-
During 24 h after LPS challenge, n	3	4	13	10			
BW, g	25.47 ± 0.99	26.13 ± 0.98	27.28 ± 0.92	26.49 ± 0.95	0.15	0.93	0.33
BW gain, g	0.57 ± 0.16	0.03 ± 0.14	1.02 ± 0.08*	0.61 ± 0.09*	< 0.05	< 0.05	0.581
Feed intake, g	2.77 ± 0.38	2.05 ± 0.33	3.32 ± 0.18	2.20 ± 0.23	0.243	< 0.05	0.502
G:F ratio, g/g	0.206 ± 0.061	0.012 ± 0.053	0.314 ± 0.037*	0.277 ± 0.029*	< 0.05	< 0.05	0.107

*Different from CON within a challenge treatment, *P* < 0.05.

^1^Values are means ± SE.

^2^CON = control diet; SDP = 8% spray-dried plasma diet; PBS = intraperitoneal injection of phosphate-buffered saline on GD 17; LPS = intraperitoneal injection of lipopolysaccharide on GD 17.

^3^D = diet effect; L = LPS challenge effect; D × L = interaction between diet and LPS challenge.

^4^Could not be calculated due to loss of BW of several mice in each treatment group.

^5^G:F ratio, ratio between daily BW gain and daily feed intake.

### Organ weight

The LPS challenge increased (*P* < 0.05) the spleen weight at 6 h after the LPS challenge and tended (*P* = 0.079) to increase it at 24 h after the LPS challenge compared with the PBS challenge, but other organ weights were not affected by the LPS challenge (Table 5). The SDP tended (*P* = 0.082) to decrease the spleen weight at 6 h, and decreased (*P* < 0.05) it at 24 h after the LPS challenge compared with the CON.

**Table 5:** Organ weights of pregnant mice^1^.

Item	Treatment^2^				*P*-value^3^		
	CON		SDP				
	PBS	LPS	PBS	LPS	D	L	D × L
At 6 h after LPS challenge, n	3	4	5	5			
BW, g	27.70 ± 0.80	25.20 ± 0.78	27.28 ± 0.76	27.28 ± 0.76	0.12	0.36	0.36
Liver wt, % of BW	4.93 ± 0.23	4.81 ± 0.20	4.68 ± 0.20	4.74 ± 0.20	0.460	0.885	0.663
Spleen wt, % of BW	0.26 ± 0.035	0.44 ± 0.031	0.26 ± 0.031	0.32 ± 0.031*	0.082	< 0.05	0.093
Lung wt, % of BW	0.56 ± 0.035	0.64 ± 0.030	0.54 ± 0.030	0.56 ± 0.030	0.114	0.163	0.321
At 24 h after LPS challenge, n	3	4	13	10			
BW, g	25.47 ± 0.99	26.13 ± 0.98	27.28 ± 0.92	26.49 ± 0.95	0.15	0.93	0.33
Liver wt, % of BW	5.17 ± 0.30	5.18 ± 0.26	5.45 ± 0.14	5.53 ± 0.18	0.198	0.854	0.874
Spleen wt, % of BW	0.22 ± 0.018	0.28 ± 0.018	0.27 ± 0.013*	0.30 ± 0.016	0.010	0.079	0.193
Lung wt, % of BW	0.54 ± 0.043	0.59 ± 0.037	0.57 ± 0.021	0.56 ± 0.026	0.991	0.497	0.363

*Different from CON within a challenge treatment, *P* < 0.05.

^1^Values are means ± SE.

^2^CON = control diet; SDP = 8% spray-dried plasma diet; PBS = intraperitoneal injection of phosphate-buffered saline on GD 17; LPS = intraperitoneal injection of lipopolysaccharide on GD 17.

^3^D = diet effect; L = LPS challenge effect; D × L = interaction between diet and LPS challenge.

## Discussion

The present study shows that the LPS challenge increased the concentrations of pro-inflammatory cytokines, TNF-α and IFN-γ, in both uterus and placenta and reduced the concentrations of anti-inflammatory cytokines, IL-10 and TGF-β1, in uterus only. These observations are in agreement with published results from previous studies [1, 2, 3, 26, 27, 28]. Results from the current experiments indicate that the LPS challenge could induce locally and systemically inflammation, as indicated by increased the production of pro-inflammatory mediators and reduced the production of anti-inflammatory cytokines. The present study also observed that the SDP reduced TNF-α, IFN-γ, and TGF-β1 in both uterus and placenta. These observations are in agreement with results from previous studies, showing that SDP alters inflammatory indicators, including pro- and anti-inflammatory cytokines [19, 29], their mRNA expressions [30, 31], or populations of immune cells [18, 32], locally or systemically [20, 21]. Therefore, the cytokine results in the present study indicate that the dietary SDP attenuates inflammation caused by LPS.

It has been reported that LPS challenge could affect animal behaviors, such as decreased feed intake, increased “slow-wave sleep”, and others [33, 34, 35], suggesting that behavioral responses can be used as indicators of inflammation and as sickness behaviors [36]. In the current experiments, we observed that LPS challenge reduced feed intake, moving time, and number of rearing, but increased lethargy score. These observations imply that the inflammation caused by LPS challenge induce abnormal behaviors. On the other hand, the dietary SDP attenuated the lethargic behaviors caused by LPS infection in the current study. These results may be attributed that the SDP reduced pro-inflammatory mediators in LPS-challenged mice. However, further research is required to investigate more detail and exact correlation between anti-inflammatory effect of SDP and attenuation of lethargic behaviors caused by LPS challenge.

The present study shows that the LPS challenge caused late-term pregnancy loss and fetal death. These results are in agreement with results from previous studies, indicating that acute inflammation caused by the LPS challenge causes the fail to pregnancy maintenance, fetal death, or both induced by the immune imbalance from the increased concentrations of pro-inflammatory cytokines in gestational tissues [1, 2, 3]. Meanwhile, feeding the SDP did not ameliorate the reproductive losses, although it attenuated the concentrations of pro-inflammatory cytokines in the gestational tissues. Maybe the attenuation of inflammation by feeding the SDP was not strong enough to increase the proportion of mice that maintained late-term pregnancy in this experimental model. On the other hand, previous studies show that intraperitoneal administration of an anti-inflammatory cytokine, IL-10, after the LPS challenge can suppress the increased concentration of pro-inflammatory cytokines and ameliorate the detrimental reproductive losses caused by the LPS 2, 3].

In the present study, the LPS challenge reduced maternal growth rate, presumably because of reduced feed intake [34] and diversion of energy and nutrients from growth to immunity [30, 37, 38]. Meanwhile, the SDP improved the growth rate. This result is in agreement with several reviews about effects of SDP in nursery pig diets [39, 40, 41]. It suggests that the SDP provides physiological benefits beyond provision of bioavailable nutrients [11]. These physiological benefits may include strengthening gut barrier function [16, 42, 43], antibacterial effects [44, 45], regulating immunity [20, 21, 29], and others in normal as well as challenging conditions. Results in the present study indicate that the SDP improves maternal growth either in normal or challenging condition.

The potential physiological benefits of the SDP may also contribute to increased fetal weight shown in the present study. However, a previous study did not show improvement of birth weight of piglets from pregnant sows fed a low level of SDP [46] and there is no corresponding information for mouse or rat. Therefore, the question of the conditions in which SDP can increase fetal weight is not resolved.

The present study shows that the LPS challenge increased spleen weight at 6 and 24 h after the LPS challenge. Organ weight, especially spleen weight, can be used as an indicator of severity of inflammation caused by the LPS challenge [47, 48]. Greater organ weight may indicate more severe inflammation as more immune cells are recruited into the organ [47, 48]. Meanwhile, the SDP reduced the spleen weight only at 6 h after the LPS challenge, perhaps because of the anti-inflammatory effects of SDP. Previously, the SDP reduced the spleen weight after *E*. *coli* challenge in one pig study [37]. The reduction of spleen weight by SDP supports other observations indicating that SDP attenuates inflammation caused by LPS.

Overall, dietary SDP attenuated inflammation and behavioral responses to LPS administration and improved fetal and maternal growth, but there is no indication that it affected late-term pregnancy loss or fetal death after the acute inflammation. Thus the reproductive benefits derived from the anti-inflammatory effects of feeding SDP may be stronger in early pregnancy [10] than in late pregnancy. To our knowledge, the present study is the first to show that dietary SDP attenuates inflammatory and behavioral responses of pregnant mice under acute inflammation caused by LPS. However, more research will be needed to decipher the benefits of feeding SDP on maternal inflammation and behaviors.
